# Resident education in radiology in Europe including entrustable professional activities: results of an ESR survey

**DOI:** 10.1186/s13244-023-01489-4

**Published:** 2023-08-22

**Authors:** European Society of Radiology (ESR), Jussi Hirvonen, Minerva Becker, Hannu J. Aronen

**Affiliations:** European Society of Radiology (ESR), Am Gestade 1, 1010 Vienna, Austria

**Keywords:** Graduate medical education, Training program, Curriculum

## Abstract

Entrustable professional activity (EPA) is a tool for comprehensively evaluating the level of confidence in resident performances across various competencies in medicine. The application of and attitudes towards EPAs in radiology across the European Society of Radiology (ESR) national institutional member societies is still to be determined. An online survey was conducted among ESR national institutional member societies to assess the current use of EPAs and other resident assessment forms among different countries.

Although the primary focus was on the use of EPAs, additional questions also addressed the adherence of training programs to the European Training Curriculum (ETC), other methods of continuous assessment, and examinations. A total of 65 responses were received from 38 countries (81% response rate among national institutional member societies). EPAs were being used in radiology in 21% of countries and planned to be used in 26%. Most responders considered EPAs suitable for radiology and, regarding the future, preferred European-level guidelines on EPAs over national or institutional levels. The majority (63%) of national training programs were reported to be similar to or following the content of the ETC, and the majority (95%) of countries rated the requirements of the European Training Curriculum (ETC) to be adequate. In conclusion, EPAs are beginning to be used in radiology resident training programs across Europe, and their use is expected to increase. There seems to be a positive attitude toward using EPAs in radiology and toward a common European framework.

**Critical relevance statement **As a result of this survey, we found positive attitudes towards using entrustable professional activities (EPA) in radiology among the institutional member societies of the European Society of Radiology (ESR).

**Key points**

• Twenty-one percent of national member societies use entrustable professional activities (EPA) in radiology.

• There is a positive attitude toward using EPAs in radiology.

• Majority of respondents preferred a common European framework for EPAs.

• Majority of radiology training curricula adhere to the European Training Curriculum (ETC).

**Graphical Abstract:**

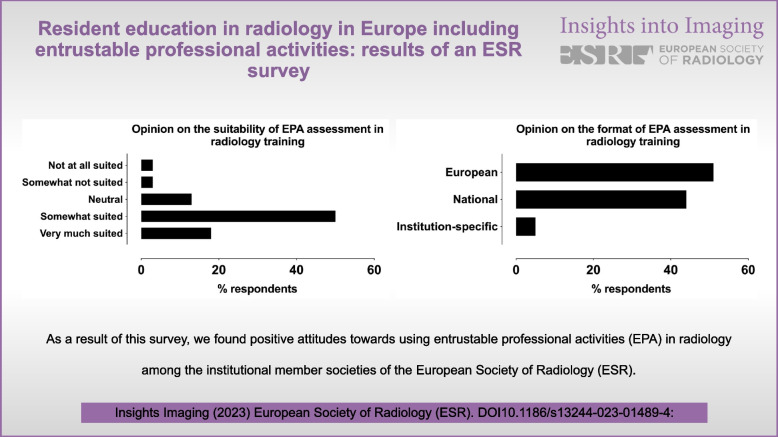

**Supplementary Information:**

The online version contains supplementary material available at 10.1186/s13244-023-01489-4.

## Introduction

The field of medical education is rapidly changing from traditional knowledge-based education to competency-based education (CBE) that complements substance knowledge with generic competencies. As a result, in addition to traditional forms of resident assessments, such as logbooks and summative examinations, entrustable professional activity (EPA) assessments are becoming popular in various medical fields. The purpose of EPAs is to complement substance knowledge with other medical competencies and to allow a more comprehensive evaluation of the confidence level in resident performance. An EPA is a set of work, or a process, that senior doctors entrust to a resident to perform independently once sufficient competence has been demonstrated (Table 1). The basic principles of EPAs have been laid out by ten Cate [1, 2], and significant progress has been made in various medical specialties [3–5].

**Table 1:** Terms and definitions used in resident education and evaluation

Abbreviation	Explanation	Definition
ETC	European Training Curriculum	A unified curriculum for radiology residency
EPA	Entrustable professional activity	Measures the level of entrustability of the work process performed by the resident
ETAP2.0	European Training Assessment Program	Evaluates the resident training program
EDiR	European Diploma in Radiology	Overall evaluation of the resident to become an independent specialist in radiology

The European Society of Radiology (ESR) has put tremendous effort into harmonizing the framework for radiological education among its member countries via the European Training Curriculum (ETC) [6]. The ETC separates competencies into Knowledge, Skills, and Competencies and Attitudes but considers EPAs only as competencies in its current form. Overall, there is limited published information on the development and deployment of EPAs in radiology [7–9], and a systematic understanding of the status of the uses of and attitudes toward EPAs among the ESR member countries is lacking. This information would be useful for member countries in their work toward developing the radiology residency curricula and deciding whether to create a common European framework for EPAs, similar to the work done toward the ETC.

A survey was conducted among the ESR national institutional member societies regarding resident evaluations in radiology to explore the landscape of various evaluation methods and to guide further collaborative efforts in harmonizing radiology training among the ESR national institutional member societies. Although the primary focus was on the use of EPAs, additional questions addressed the adherence of training programs to the ETC, other methods of continuous assessment, examinations, and interest in the European Training Assessment Program (ETAP2.0).

## Methods

An online survey (Additional file 1) was sent out to the national radiological societies of 47 ESR National Institutional Member Societies via email on April 13, 2022. A first reminder was sent via email on April 26, 2022, and a second on May 17, 2022. The survey was closed on May 23, 2022. Some contradictory results from participants in the same country were verified individually by recontacting relevant member societies and checking national requirements published by the respective governments or respective national societies. The results were then reviewed anonymously and expressed as percentages of all respondents. An average value is presented in cases of multiple conflicting responses from a single country.

## Results

### Response rate and background information on residency programs

A total of 65 responses were received from 38 countries (81% response rate among national member societies). The majority of countries (61%) reported 5 years of radiology in the residency programs, followed by 4 years (21%) and 3 years (13%) of radiology.

### Structure and assessment of residency programs

**Fig. 1:**
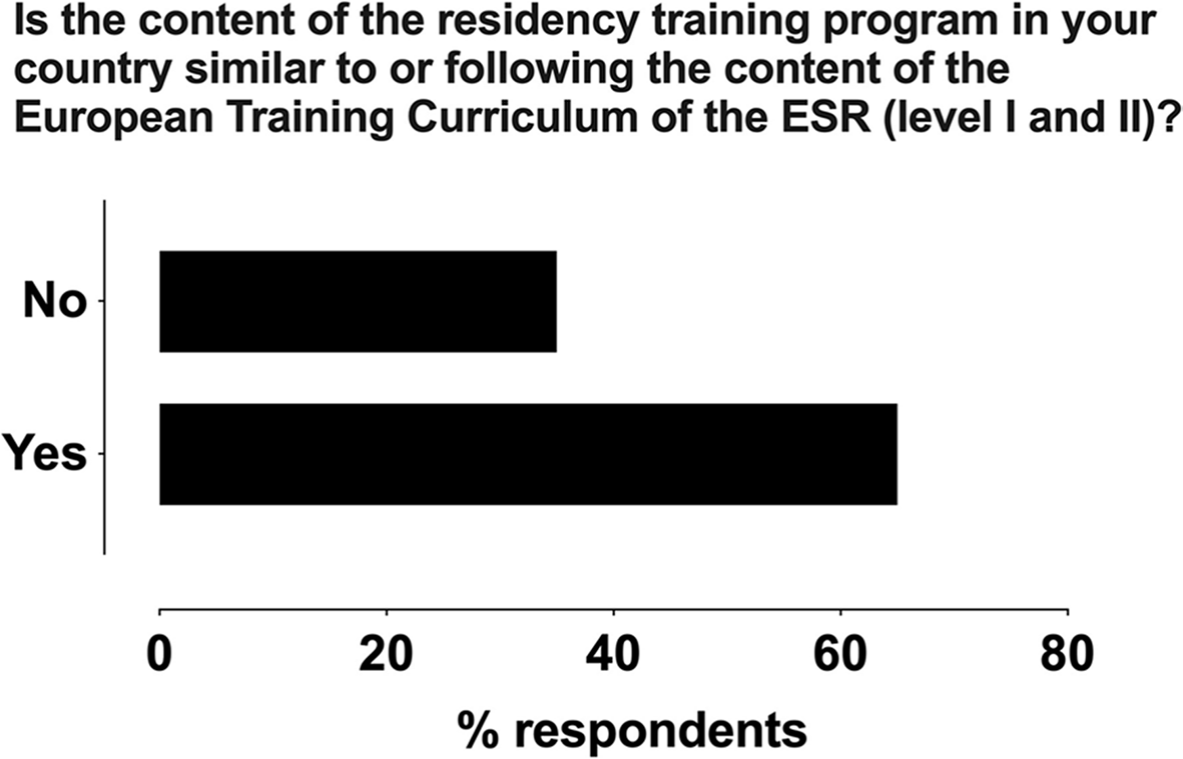
Responses on the similarity of the residency program content to the European Training Curriculum (ETC) of the ESR among those who responded (37 out of 38)

The majority (63%) of national training programs were reported to be similar to or following the content of the ETC, sometimes with minor modifications (Fig. 1). Overall, the requirements of the ETC were mostly considered adequate (95%), with some respondents reporting these requirements as too demanding (5%). Residency training programs were most often assessed by national authorities (66%) or national radiological societies (11%). Many respondents (32%) reported interest in applying for the ETAP 2.0 certification.

### Resident assessments: entrustable professional activity (EPA)

A total of 21% of the respondents reported that EPAs are currently being used in their residency programs, whereas 26% of the countries reported that the introduction of EPAs was being planned in either all or at least some training institutions in the next 10 years. A national requirement to implement EPA assessments in other specialties than radiology was reported by 26% of country representatives.

**Fig. 2:**
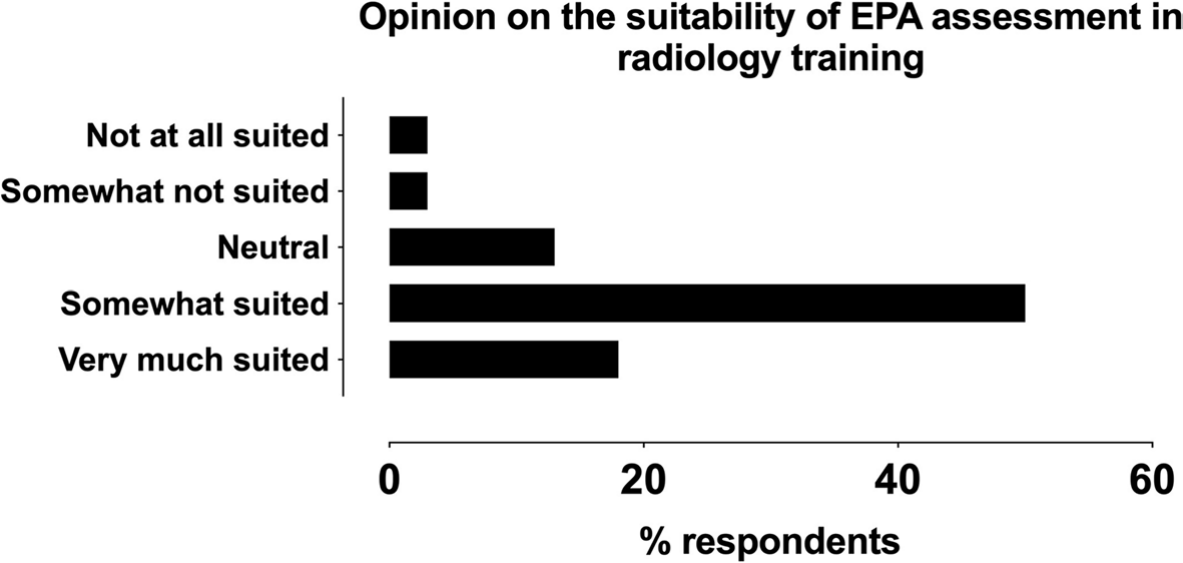
Responses on the perceived suitability of EPAs in radiology, excluding those that did not respond (13%)

**Fig. 3:**
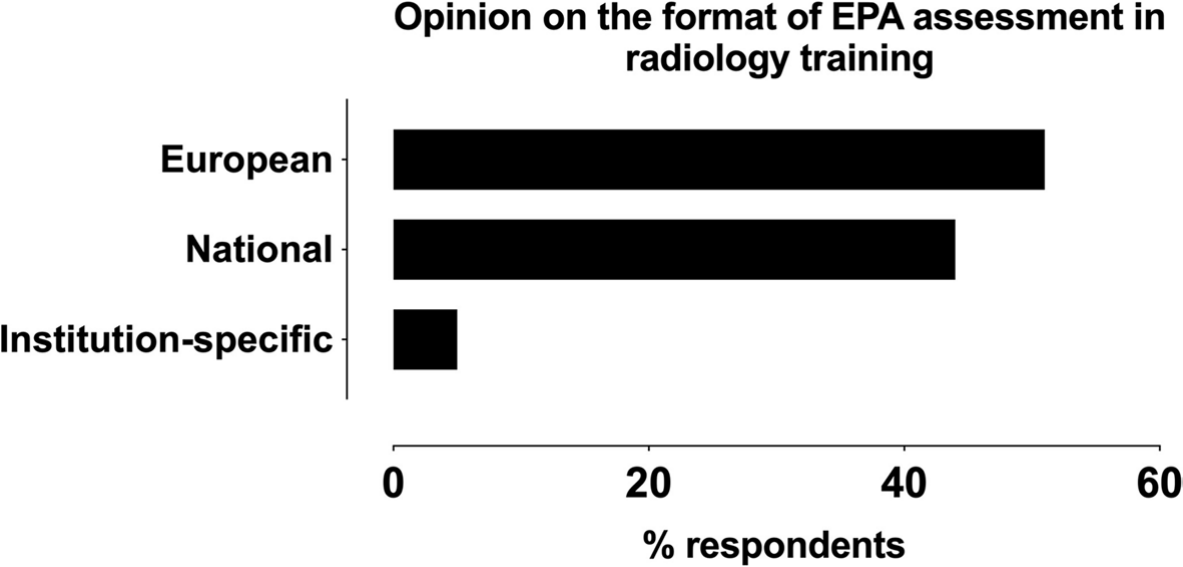
Responses on the preference of format of EPAs in radiology

Regarding attitudes towards EPA assessments, 69% of countries considered EPAs either well or very much suited for radiology, 13% considered them moderately well suited, whereas a minority of 5% considered them not suited (13% did not respond) (Fig. 2). About half of the respondents (51%) felt that if EPA assessments were to be a regular part of radiological residency training, they should be defined on a European level, whereas 44% supported national EPAs, and 5% supported institution-specific assessments (Fig. 3).

### Resident assessments: logbooks, exams, etc.

Regarding the continuous assessment of residents, written and/or oral examinations (77%) and on-site evaluations by tutors (64%) were the most commonly used methods, followed by evaluations by more senior residents (13%). Some respondents reported nationwide web-based assessments or annual faculty assessments and feedback from all colleagues (senior, junior, and non-medical). Most countries (66%) reported a nationally required board examination to become a radiologist. About 96% of these board examinations were separated between theory exams and image interpretation exams, and about half had additional radiation protection exams. A logbook was required in 82% of countries; among these, 52% reported national coordination of logbooks, 45% reported region/hospital-based, and 3% did not specify.

## Discussion

A representative sample of responses from ESR national institutional member societies provides a unique opportunity to assess the European landscape of radiology resident assessments. These results show encouraging evidence for the adherence of national radiology training curricula to the contents of the ETC as proposed by the ESR and positive attitudes toward developing a common European framework for EPAs. About a fifth of countries used EPAs in their radiology residency programs, and about a quarter foresaw the implementation of EPAs in the future. Thus, using EPAs (21%) in the national residency curricula is currently less prevalent than adherence to the ETC guidelines (63%) provided by the ESR. Most respondents felt that EPAs are well suited for radiology, and the majority preferred European standards for EPAs. This calls for discussion about the potential contents of European EPAs for radiology and their relationship with the ETC.

An important question regarding EPAs is whether they should be generic (e.g., writing informative reports, behaving professionally) or specific to a certain task (e.g., performing ultrasound-guided biopsies). The ETC already covers some of the more generic aspects in the *Competences and Attitudes* sections of each subspecialty. This would suggest that if European EPAs are being formulated, they should span multiple subspecialties and be generic.

An issue to consider is the increased burden of EPA evaluations on radiology educators regarding workload. Artificial intelligence methods might help provide solutions for the future evaluation of imaging interpretation processes. For example, such methods might ensure that the resident sees relevant and representative cases or that the residents’ reports are of acceptable quality [10]. These technological advances in radiological education are not limited to EPAs but could be used in more traditional forms of assessments, such as logbooks. Nevertheless, evaluating practical skills, e.g., performing an ultrasound examination of the thyroid gland or a biopsy, contributing to decisional multidisciplinary conferences, or assessing a radiologist’s communication skills with patients or colleagues from other disciplines, are still the domain of evaluations by experienced educators.

Reporting from the USA, Deitte et al. published results from discussions by a multi-institutional work group regarding EPAs initially developed by the Accreditation Council for Graduate Medical Education (ACGME) [7]. This group identified ten EPAs for radiology: (1) collaborates as a member of an interprofessional team; (2) triages/protocols exams; (3) interprets exams and prioritizes a differential diagnosis; (4) communicates results of exams; (5) recommends appropriate next steps; (6) obtains informed consent and performs procedures; (7) manages patients after imaging and procedures; (8) formulates clinical questions and retrieves evidence to advance patient care; (9) behaves professionally; and (10) identifies system failures and contributes to a culture of safety and improvement [7]. These EPAs are generic because they can be applied to various subspecialties and combine multiple core competencies.

Another report from the USA by Sheth et al. [9] identified five EPAs specifically for breast radiology (with eight nested EPAs and one elective): (1) identifying and managing abnormalities on screening examinations; (2) working up and managing patients in the diagnostic imaging setting; (3) performing biopsies using imaging guidance and determining appropriate postprocedural management; (4) evaluating and staging patients with newly and previously diagnosed breast cancer, and (5) performing presurgical localization using ultrasound or mammographic guidance. This is an example of a subspecialty set of EPAs that is tailored to the specific needs of breast radiology.

In the Netherlands, Graafland et al. surveyed seven medical specialties, including radiology, to identify EPAs that would be considered important in the specialty’s residency training [8]. Respondents from radiology identified the following EPAs as important: (1) ultrasound-guided mammarian puncture; (2) ultrasound-guided biopsy; (3) ultrasound-guided intra-abdominal abscess drainage; (4) management of trauma patients; (5) assessment of chest X-ray; (6) angiographic intervention radiology; (7) arthrography; (8) ultrasonography (diagnostic); and (9) lead multi-disciplinary planning of intervention [8]. These are somewhat more specific than those proposed by Deitte et al. [7], but the lack of various tasks, techniques, and imaging targets suggests that this collection of EPAs was not designed to be exhaustive.

Nayyar et al. [11] developed a set of six EPAs with 87 competencies and assessment strategies for undergraduate studies in radiology in Pakistan. These EPAs were rather broad in definition, such as “Recognize, interpret, and communicate results of common pathologies and emergencies on chest radiographs.” While these EPAs intended for undergraduate clerkships are not likely applicable to radiology residency curricula, the process of developing EPAs via expert consensus may be informative.

Interpretation of the results from our survey study may be limited by sampling and responder bias. Yet, we had a reasonably high response rate among ESR national institutional member societies (81%). Different countries may have different conceptions and expectations of EPAs, and this issue warrants future discussion and consensus among the ESR national institutional member societies.

The requirements of the ETC were mostly considered adequate (95%), with some respondents reporting these requirements as too demanding (5%). This indicates that although the requirements of ETC have considerably increased during the last ten years, the position of ETC as a normative basis for radiological resident training in Europe is widely accepted. Since 2012, the ETC has been in a form consistent with The European Union of Medical Specialists (UEMS) standards, and the document has been endorsed by all the national Societies and the UEMS Radiology Session. In our survey, a considerable portion of the responders preferred European EPAs. If European EPAs are developed, it would be important to bind them strictly to the requirements of ETC. European EPAs could be an additional tool in the harmonization process of European radiological education. A structural evaluation system for European EPA would be needed to be established beforehand, separate from the development process of EPAs. Naturally, the involved cost, organizational issues, and the availability of the necessary professional expertise would have to be examined.

Although the main focus of the current study was EPAs, we included ETC because of its central role in harmonizing radiology education in Europe. The results of the present survey confirm that in most European countries, the duration of the resident program is 5 years, according to the recommendations of ETC. The ETC and ETAP program has helped national societies and university hospitals to lengthen the duration of a national resident program into 5 years in many countries [12]. The curriculum, including 5 years of radiology, improves the capability of the training to fulfill the extended requirements of the present ETC.

In conclusion, EPAs are beginning to be used in radiology resident training programs across Europe, and their use is expected to increase. There is a positive attitude toward using EPAs in radiology and toward a common European framework.

### Supplementary Information

**Additional file 1. **Online survey. 

## Data Availability

All relevant data is within the manuscript.

## Abbreviations

ACGME: Accreditation Council for Graduate Medical Education
CBE: Competency-based education
EDiR: European Diploma in Radiology
EPA: Entrustable professional activity
ESR: European Society of Radiology
ETAP2.0: European Training Assessment Program
ETC: European Training Curriculum
UEMS: The European Union of Medical Specialists
