# Supplementary material for: Resident education in radiology in Europe including entrustable professional activities: results of an ESR survey
Source: Insights Imaging. 2023 Aug 22;14:139. doi: 10.1186/s13244-023-01489-4 (PMC10444922; doi:10.1186/s13244-023-01489-4)
Supplement: Supplementary file 1 — Additional file 1. Online survey. [file 13244_2023_1489_MOESM1_ESM.pdf]

Resident education in radiology in Europe including entrustable professional activities: results of an ESR survey

ELECTRONIC SUPPLEMENTARY MATERIAL

A brief questionnaire on residency training requirements at the national level in the ESR member countries

1. How many years of radiology are there in the residency training program of your country?

- 3 years
- 4 years
- 5 years
- Other

2. Does the residency training program of your country specify whether the radiology training has to take place in

- only one hospital
- more than one hospital
- does not specify

3. Does the residency training program require a logbook?

- Yes
- No

If yes,

- is the logbook national?
- is the logbook hospital-based?
- is the logbook region-based

4. Is the content of the residency training program in your country similar to or following the content of the European Training Curriculum of the ESR (level I and II)?

- Yes
- No

If yes, how is the European Training Curriculum (level I and II) content used?

- Open question: free text

5. In the present version of the European Training curriculum of the ESR (level I and II), are the requirements:

- Too demanding?
- Adequate?
- Too easy?

6. How is the continuous assessment of residents accomplished in your country?

- Written and/or oral examinations
- On site evaluations by the tutors
- Evaluations by older residents
- Other (free text specify)

7. Who assesses the residency training programs in your country?

- National authorities
- ETAP
- National Radiological societies
- Other
- Not assessed

8. Do you currently use Entrustable Professional Activity (EPA) assessments in radiology in your country?

- Yes
- No

9. Are Entrustable Professional Activity (EPA) assessments in radiology planned to be introduced in your country in the next 10 years?

- Yes, in all institutions
- Yes, in some institutions
- No

10. If EPA assessments will be a part of the resident training of radiology in Europe, the EPAs should be:

- Institution-specific
- National
- European

11. Is there currently a national requirement to implement EPA assessment across other specialties than radiology in your country?

- Yes
- No

12. What is your opinion about the suitability of EPA assessments in radiology on a scale from 1 (not at all suited) to 5 (very much suited)?

- 1
- 2
- 3
- 4
- 5

13. Have training centers in your country considered applying for certification by the European Training Assessment Program 2.0 (ETAP2.0)?

- Yes
- No

14. Do you have a nationally required board examination to become a radiologist?

- Yes
- No

If yes, specify if yours has a separate:

Theory exam

- Yes
- No

Imaging interpretation exam

- Yes
- No

Radiation protection exam

- Yes
- No
